# Women in Chemistry: Q&A with Dr Shira Joudan

**DOI:** 10.1038/s42004-024-01303-2

**Published:** 2024-10-04

**Keywords:** Atmospheric chemistry, Analytical chemistry

## Abstract

Dr Shira Joudan is an Assistant Professor in the Department of Chemistry at the University of Alberta in Edmonton, Canada. Her environmental analytical chemistry research group studies the environmental fate of organic contaminants, including halogenated chemicals like per- and polyfluoroalkyl substances (PFAS).

**Figure:**
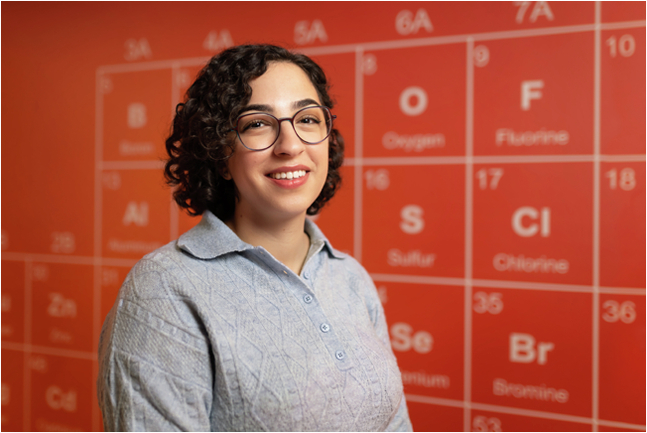
Dawn Graves

Shira’s research group is specifically interested in how chemical reactions in the environment impact how contaminants are transported and potentially accumulate. To do this, the group innovates chromatography and mass spectrometry-based analytical methods to apply to environmental samples and lab experiments. Ultimately, their research helps inform policy and hopefully helps to design chemicals that provide functionality without environmental harm. Shira grew up in Winnipeg, obtained her BSc from Carleton University, her PhD from the University of Toronto, and held a postdoctoral research position at York University in Toronto. Outside of research and teaching, she is a columnist for *Nature Chemistry*, where she writes about being a pre-tenure faculty member, and enjoys exploring cities, live music, and spending time with friends.

Why did you choose to be a scientist?

I did not always know I would be a scientist, although I have always been curious about how things work. In school, I liked my science classes and being outdoors, which led me to B.Sc. In a step-wise path of opportunities, including a few small pivots, I realized how much I enjoyed my Ph.D. research and my specific research topic. During my postdoc, I realized that pursuing an academic career as a scientist would be a fulfilling way to pursue my interests.

What scientific development are you currently most excited about?

I am really excited about how new analytical instrumentation and data handling methods allow us to better understand the fundamental chemistry that occurs in the environment. For a long time, researchers have done their best with the tools available, but to me, it really feels like now is an amazing time to push our understanding with higher throughput experimentation and better measurements with improvements like faster online sampling or lower detection limits.

What direction do you think your research field should go in?

I think the field of environmental chemistry needs to continue to perform high-quality research to inform policy and risk assessment and the production of greener chemicals. A lot of research findings are extrapolated beyond the scope of the initial study, and this can have serious consequences with public trust. I think it is critical to ensure we are performing the experiments that answer specific questions and effectively communicating their limitations.

What topics do you most enjoy teaching?

I teach undergraduate environmental and analytical chemistry courses. For environmental chemistry, I think having undergraduates learn about chemical processes in the environment helps them to understand the potential impact of what they are doing in the lab. And, in today’s world of climate change and chemical pollution, I think its powerful to have over 100 students a year leave my course with the ability to help to dispel misinformation because they understand the fundamental chemical changes occurring in the environment. For analytical, I really believe that teaching students how to make meaningful measurements is key to any future scientific problem they address, including being confident (or skeptical) about data.

What do you most (and least) enjoy about being a scientific researcher?

I love that, even briefly, you may be the only person in the world that knows something. Since becoming a professor, what I love most has changed to my students excitedly sharing their new observation with me, so maybe I am more often the second or third person to know something now. As for what I least enjoy, I do not love the paperwork and bureaucracy that come with the job.

Have you been a minority as a woman at any stage of your career? What was that experience like for you?

During my Ph.D., there were a lot of women grad students, and despite there being few women professors, it felt like things could be changing. It was not until starting as a professor that I felt I was truly a minority in terms of gender. Something I did not expect is how often my being a woman professor in chemistry is brought up as academia tries to reckon with its sexism and other discrimination. As someone who would prefer to not think of their gender often, it is something I am adapting to, and figuring out how to speak up while protecting myself. Most comments are positive, such as ensuring women are represented as seminar speakers, but it has been shocking to see what some people say behind closed doors now that I am in more powerful rooms.

How can individual scientists support and celebrate their women colleagues?

Nominate women for awards that are not specifically designated for women in science. Reflect on who you invite into collaborations or exclusive events – if there are no women (or if the group is homogenous in other ways), reflect on why that is. And make sure that your feminism is intersectional because not all women (or gender diverse people!) are the same.

Are you or have you been supported by a mentor? What was the best advice you received?

Mentors have been key to my success and happiness, and my top recommendation is to find your people and keep them close. Some mentors have been assigned formally, while others are relationships I have developed over time either through my Ph.D. supervisor’s network of alumni, or people I have met at conferences. Of the advice I’ve received I most resonate with the following:

1. Do something you find interesting.
2. You cannot do it all, so be selective.
3. If you are doing something novel that has not been done before, it is ok if it is not perfect so long as you can clearly understand and explain the limitations.
4. Not everyone will like you or your work, so it is best to try to accept that.

Where do you hope to see women in chemistry in 20 years?

I hope that women, and all chemists, can bring as much of themselves to their job as they would like to, and ideally, we would not need to be actively fighting against systemic and individual experiences of sexism.

*This interview was conducted by the editors of Communications Chemistry*.

